# Prophylactic application of laser light restores L-FABP expression in the livers of rats submitted to partial ischemia

**DOI:** 10.6061/clinics/2018/e113

**Published:** 2018-06-19

**Authors:** Kelvin Henrique Vilalva, Rebeca Lopes Figueira, Marina Silveira, Catarina Graf, Frances Lanhellas Gonçalves, Lourenço Sbragia, Maria Cecília Gomes, Fabrícia Mumic, José Dirceu Vollet-Filho, Vanderlei Salvador Bagnato, Luiz Augusto Carneiro D’Albuquerque, Orlando Castro-e-Silva

**Affiliations:** I Divisao de Transplante de Figado, Departamento de Cirurgia e Anatomia, Faculdade de Medicina de Ribeirao Preto, Universidade de Sao Paulo, Ribeirao Preto, SP, BR; II Laboratorio de Cirurgia Fetal Experimental, Divisao de Cirurgia Pediatrica, Departamento de Cirurgia e Anatomia, Faculdade de Medicina de Ribeirao Preto, Universidade de Sao Paulo, Ribeirao Preto, SP, BR; III Departamento de Fisica e Ciencia dos Materiais, Instituto de Fisica de Sao Carlos, Universidade de Sao Paulo, Sao Carlos, SP, BR; IV Departamento de Gastroenterologia, Faculdade de Medicina FMUSP, Universidade de Sao Paulo, Sao Paulo, SP, BR

**Keywords:** Ischemia, Reperfusion Injury, Laser Therapy, L-FABP

## Abstract

**OBJECTIVES:**

The objective of the present study was to evaluate the protective effect of pre-conditioning treatment with laser light on hepatic injury in rats submitted to partial ischemia using mitochondrial function and liver fatty acid binding protein as markers.

**METHODS:**

Rats were divided into four groups (n=5): 1) Control, 2) Control + Laser, 3) Partial Ischemia and 4) Partial Ischemia + Laser. Ischemia was induced by clamping the hepatic pedicle of the left and middle lobes of the liver for 60 minutes. Laser light at 660 nm was applied to the liver immediately prior to the induction of ischemia at 22.5 J/cm^2^, with 30 seconds of illumination at five individual points. The animals were sacrificed after 30 minutes of reperfusion. Blood and liver tissues were collected for analysis of mitochondrial function, determination of malondialdehyde and analysis of fatty acid binding protein expression by Western blot.

**RESULTS:**

Mitochondrial function decreased in the Partial Ischemia group, especially during adenosine diphosphate-activated respiration (state 3), and the expression of fatty acid binding protein was also reduced. The application of laser light prevented bioenergetic changes and restored the expression of fatty acid binding protein.

**CONCLUSION:**

Prophylactic application of laser light to the livers of rats submitted to partial ischemia was found to have a protective effect in the liver, with normalization of both mitochondrial function and fatty acid binding protein tissue expression.

## INTRODUCTION

The hepatic ischemia/reperfusion (HIR) process has serious local and systemic repercussions, resulting in high morbidity and mortality rates, mainly among surgical patients 1.

Laser light therapy, or photobiostimulation, has been used for decades in the treatment of lower limb ulcers, vascular neoformation, fibroblast proliferation, nerve regeneration and even tumors. Its efficacy in hepatic treatment has been demonstrated in several experimental studies on rats submitted to partial resection or liver transplantation 2.

Laser light is assumed to cause conformational changes in the cytochromes of hepatocytes, leading to a probable increase in adenosine triphosphate (ATP) production, which results in an increase in the energy metabolism of the liver.

In recent years, investment in studies on pre-conditioning treatments has increased. In pathological conditions or surgical procedures, where tissue stress is already expected, stimulating the tissue with small cycles of stress results in less pain, inflammation and edema 3. Several modalities, such as ischemic pre-conditioning, hyperbaric chambers and hypothermia, are already well-known and have been extensively studied in the literature. However, little information is available regarding pre-conditioning with laser light, and even less information is available regarding its preventive action on hepatic ischemic damage 4-6.

Liver fatty acid binding protein (L-FABP) is a constitutive cytoplasmic protein responsible for the transport of long-chain fatty acids that is found in the non-injured liver. Recently, L-FABP has been reported to be an effective marker for the detection of liver damage and has been investigated as a potential candidate for the detection of hepatocyte death or rupture. Because it is a low molecular-weight protein (15 kDa), L-FABP is easily detected in the plasma and urine after tissue damage 7. The use of L-FABP in the early diagnosis of various diseases of the digestive system or in surgical procedures is considered an effective method for preventing further organ damage. The main investigations available in the literature refer to inflammatory and infectious diseases, such as necrotizing enterocolitis, renal dysfunction and kidney transplantation 8-11.

Therefore, our objective was to evaluate the protective effect of pre-conditioning treatment with laser light on hepatic injury in rats submitted to partial ischemia using L-FABP as a marker.

## MATERIALS AND METHODS

### Animals

The project was approved by the Ethics and Animal Experimentation Committee of the Ribeirão Preto Medical School, University of São Paulo. Adult male Wistar rats were maintained under controlled conditions of light (12 hours of light/12 hours of dark), temperature (average 23°C) and relative humidity (close to 55%) and had free access to water and rations for rodents. All procedures involving animals were approved by the Ethics Committee on Animal Experimentation of the Ribeirão Preto Medical School (CETEA – FMRP-USP), protocol number 014/2006.

### Formation of Experimental Groups

The rats were divided into four groups (n=5 per group): 1) Control (C), 2) Control + Laser (CL), 3) Partial Ischemia (PI) and 4) Partial Ischemia + Laser (PIL).

### Anesthesia, Induction of Ischemia and Sample Collection

All animals were submitted to general anesthesia. The drugs used were xilazin, 20 mg/ml (Dopaser®, Gepec, Brazil), and ketamine, 50 mg/ml (Ketalar® Pfizer, Brazil). The dose was 15 units/100 g of weight via intraperitoneal administration.

The control group was submitted to the same surgical procedure of median laparotomy as the experimental groups with liver exposure, but not the ischemia procedure. After 90 minutes, the liver and blood were harvested.

For the induction of partial ischemia (PI), a median laparotomy with liver exposure was performed, followed by hepatic pedicle clamping of the left and middle lobes of the liver for 60 minutes. Laser light was applied to the liver prior to the induction of PI at 22.5 J/cm^2^. The animals were sacrificed after 30 minutes of reperfusion, for a total of 90 procedural minutes. Blood and liver tissues were collected for biochemical and molecular biology analyses.

### Laser Application

This study used a BW 660, class III b LASER apparatus with continuous emission of visible light on low power, with an active semiconductor medium and a transducer of fused optical fibers of 80 mm in length and 7 mm in diameter, produced by M.M. Optics LTDA (São Carlos, SP, Brazil). The wavelength of choice was 660 nm for high penetrability through biological tissue, regulated at a power output of 30 mW for a 0.04-cm^2^ irradiation probe (for a total of 750 mW/cm^2^ irradiance), with an irradiation dose of 22.5 J/cm^2^ for 30 seconds at 5 points, resulting in a total dose of 112.5 J/cm^2^. The five points of application were: two points in the right lobe (subdivided: 1 in the anterior lobule and 1 in the posterior lobule), one point in the median lobe, one point in the left lobe and one point in the caudate lobe.

### Mitochondrial Function

The parameters studied were respiration and membrane permeability of mitochondria isolated from hepatic tissue. For mitochondrial preparation, a Potter-Elvehjem-type homogenizer was used in medium containing 250 mM sucrose, 1 mM EGTA and 10 mM Hepes-KOH with a pH of 7.2. Mitochondria were isolated by differential centrifugation at 4°C 12, and mitochondrial proteins were determined by the Coomassie method (Coomassie plus - Bradford Assayt kit) (Pierce Biotechnology, Road Rockford, IL-USA) 13. Oxygen consumption was measured polarographically at 30°C using an Oxytherm (Hansatech Instruments, Norfolk, United Kingdom) in respiration medium containing 125 mM sucrose, 65 mM KCl, 10 mM Hepes, 1 mM MgCl2, 2 mM KH2PO and 0.1 mM EGTA with a pH of 7.4, and 2.0 mg/ml of mitochondrial protein energized with 5 mM potassium succinate. State 3 corresponds to oxygen consumption coupled to ATP synthesis by the addition of 200 nmol of adenosine diphosphate (ADP) and is therefore called activated respiration. On the other hand, state 4 represents oxygen consumption after consumption of the added ADP, which is a basal measure of mitochondrial bioenergetic metabolism. The measurements of states 3 and 4 are expressed as *n* oxygen atoms/min/mg of mitochondrial protein. The respiratory control ratio (RCR) was calculated as the ratio between the rate of oxygen consumption in state 3 and the rate of oxygen consumption in state 4. Alteration of the RCR indicates the degree of coupling between oxygen consumption and the synthesis of ATP by mitochondria 14.

### Determination of Mitochondrial Osmotic Swelling (Swelling)

The transition of internal mitochondrial membrane permeability induced by 20 μM calcium chloride and 1 mM potassium phosphate was determined at 540 nm in a Beckman DU-640B spectrophotometer (GMI - Ramsey, MN-USA) by reducing the optical density (ΔDO). The results are expressed as absorbance variation (Δ Abs) 15.

### Determination of Malondialdehyde (MDA)

Malondialdehyde is one of the final products derived from the peroxidation of fatty acids, and its determination represents a convenient measure of lipid peroxidation. Colorimetric determination of MDA was performed in the liver. A reaction with thiobarbituric acid was carried out at 532 nm on a Versamax (Molecular Devices-LLC, San Jose, CA- USA) microplate reader using 1,1,3,3-tetramethoxypropane (0 to 100 μM) as the standard, and the results are expressed as μM/mg of protein 16.

### Serum Aminotransferases

 serum levels of the enzymes alanine aminotransferase (ALT) and aspartate aminotransferase (AST) using the kinetic method at 340 nm with a commercial spectrophotometer (CELM SB190 - Celmrm®, Barueri, SP-Brazil) as described by Henry et al. 17.

### Western Blotting Analysis of L-FABP

After homogenization of the liver, aliquots containing 20 μg of protein from each group were added to 12% polyacrylamide gel containing 0.1% sodium lauryl sulfate (SDS-PAGE) and run buffer. Electrophoretic separation of the proteins was performed at a constant current intensity (100 V) for approximately 2 hours. Subsequently, the protein bands were electrophoretically transferred to a nitrocellulose membrane in 120-V transfer buffer at 4°C for 90 minutes. Nonspecific binding sites of the primary antibody to the membrane were blocked by incubating the membrane in 5% skim milk solution in 0.01 M PBS buffer at a pH of 7.4 under constant stirring for one hour. The membranes were then incubated for 12 hours at 4°C with rabbit anti-FABP-L primary antibody (sc-50380, Santa Cruz Biotechnology, CA, USA) diluted 1:1000 in PBS/3% BSA. After incubation, the membrane was washed with 0.01 M PBS buffer at a pH of 7.4 and incubated with anti-rabbit secondary antibody (sc-2768, Santa Cruz Biotechnology, CA, USA) diluted 1:2000 in PBS/3% BSA for two hours. The membrane was then visualized by chemiluminescence for 5 minutes. Membranes were developed using the ChemiDoc™ XRS + System Photodocumentation System (Bio-Rad Laboratories, Alfred Nobel Drive, Hercules, CA, USA), and bands were analyzed and measured with Image Lab™ (Media Cybernetics Inc., Rockville, MD, USA).

### Statistical Analysis

Analysis of Variance (ANOVA) followed by the Tukey-Kramer post-test was used for the data analysis, with the level of significance set at *p*<0.05. The results were expressed as the mean ± standard deviation. Calculations were performed using GraphPad Prism 5.0 software (GraphPad Software Inc., La Jolla, CA, USA).

## RESULTS

### Mitochondrial Function

**Figure 1:**
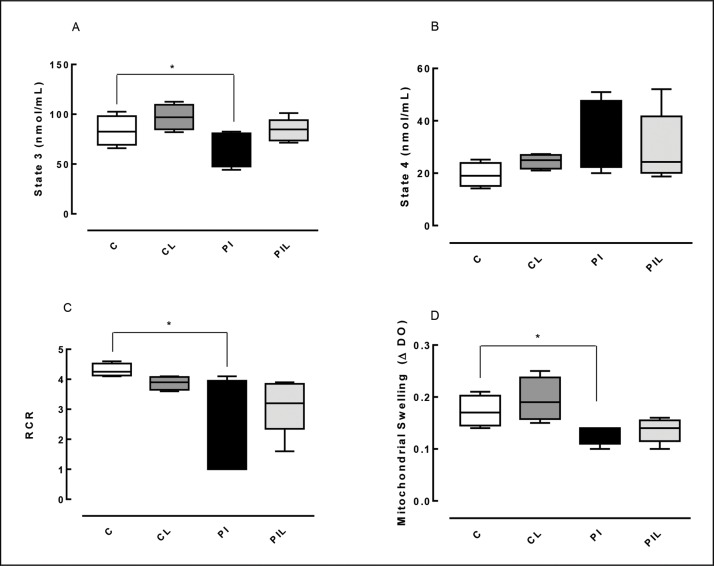
Mitochondrial bioenergetic evaluation. C: Control; CL: Control + Laser; PI: Partial ischemia; PIL: Partial ischemia + Laser. Panel A) ADP: Activated Oxygen Consumption Velocity (state 3); Panel B) Basal Oxygen Consumption Rate (state 4); Panel C) RCR: Respiratory Control Ratio; Panel D) Mitochondrial Swelling. *p<0.05.

A decrease in state 3 (the rate of oxygen consumption in the activated state) was observed in the PI group (Partial Ischemia) compared to that in the C group (Control) [PI *vs.* C (*p*<0.05)]. No statistically significant difference was identified between the C and Partial Ischemia + Laser (PIL) groups [PIL *vs.* C (*p*>0.05)]. For state 4 (basal mitochondrial respiration), no statistically significant difference was identified between the groups studied. The RCR was similar to state 3: PI *vs.* C (*p*<0.05) (Table 1) (Figure 1).

### Mitochondrial Swelling

Significant differences were observed between the PI and C groups (*p*<0.05) and the PIL and C groups (*p*<0.05) (Table 1) (Figure 1).

### MDA (Malondialdehyde)

**Figure 2:**
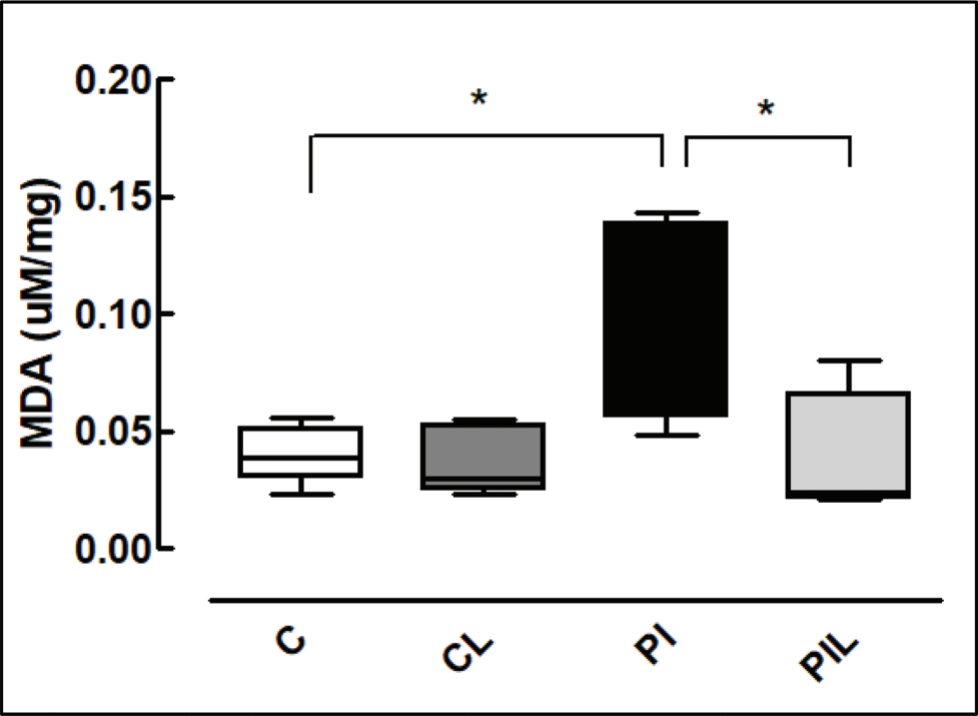
Determination of malondialdehyde (MDA). C: Control; CL: Control + Laser; PI: Partial Ischemia; PIL: Partial Ischemia + Laser. *p<0.05.

A statistically significant difference in MDA was found between the Partial Ischemia group (PI) and the other groups (*p*<0.05) (Table 1) (Figure 2).

### Serum Aminotransferases

**Figure 3:**
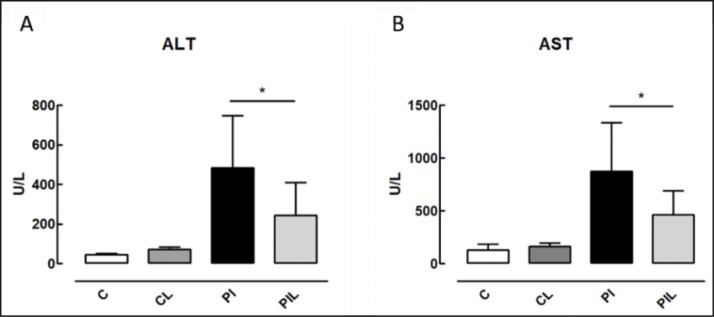
Serum aminotransferases. Panel A) ALT: alanine aminotransferase. Panel B) AST: aspartate aminotransferase. C: Control; CL: Control + Laser; PI: Partial Ischemia and PIL: Partial Ischemia + Laser. *p<0.05.

The ALT and AST enzyme measurements revealed significant differences between the PIL and PI groups (*p*<0.05), with the PIL group showing decreased levels of these enzymes. The levels in the C and CL groups were reduced compared with those in the PI and PIL groups (**p<0.005). In Figure 3, we show the differences between the PI and PIL groups.

### L-FABP Analysis by Western Blot

**Figure 4:**
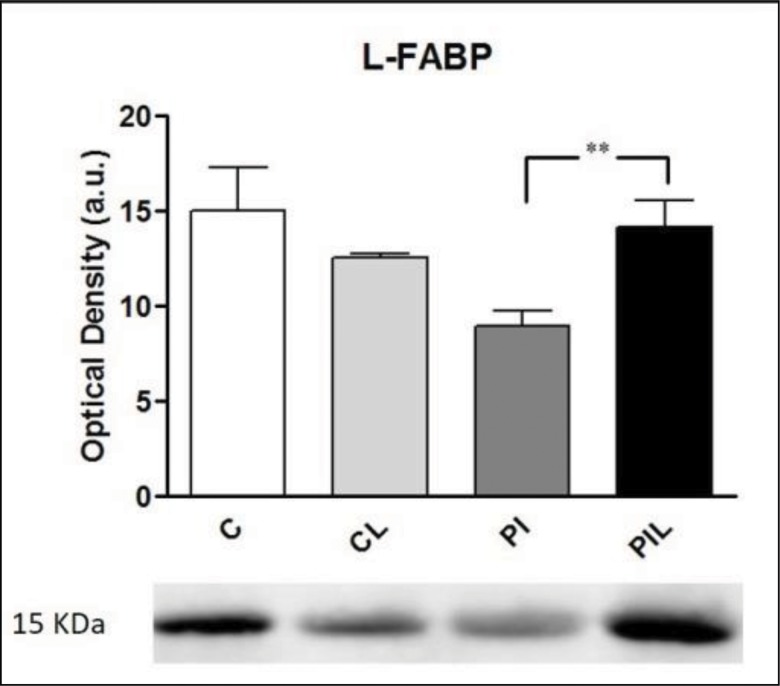
L-FABP expression, showing protein detection at 15 kDa (below) and the optical density values (above). C: Control. CL: Control + Laser. PI: Partial Ischemia. PIL: PI + Laser. au: arbitrary unit. (**p<0.005).

The evaluation of L-FABP expression by Western blot revealed decreased expression in the PI group compared to that in groups C, CL and PIL (*p*<0.005) (Figure 4). Optical density values, in arbitrary units, were (n=5): C=15.04 (±2.25); CL=12.58 (±0.15); PI: 8.92 (±0.83); and PLI: 14.12 (±1.44). Comparisons between groups: CxCL: [15.04 (±2.25) *vs.* 12.58 (±0.15) (NS)]; CxPI: [15.04 (±2.25) *vs.* 8.92 (±0.83) (*p*<0.005)]; CxPLI: [15.04 (±2.25) *vs*. 14.12 (±1.44) (NS)]; CLxPI: [12.58 (±0.15) *vs*. 8.92 (±0.83) (*p*<0.05)]; CLxPLI: [12.58 (±0.15) *vs*. 14.12 (±1.44) (NS)]; PIxPLI: [8.92 (±0.83) *vs*. 14.12 (±1.44) (*p*<0.005)].

## DISCUSSION

Ischemia/reperfusion (IR) is a phenomenon characterized by cellular damage after a period of hypoxia followed by reperfusion 18. Hepatic ischemia/reperfusion (HIR) is a complex process that can gravely impair liver function and represents a systemic process that affects multiple tissues, thus promoting a cascade of multiple organ dysfunction 19.

Several mechanisms are linked to HIR, including reactive oxygen species (ROS) generation, alteration of calcium homeostasis, lipid peroxidation, mitochondrial dysfunction, activation of liver Kupffer cells (KCs) and cytokine production 20. The functional integrity of the liver after injury can be determined indirectly by the mitochondrial function of the tissue, i.e., by the ability of the previously injured liver to synthesize energy 21. The present study evaluated mitochondrial respiration based on the oxygen consumption rate in state 4 (basal respiration), the oxygen consumption rate in state 3 (respiration activated by ADP) and the RCR, which indicates the degree of coupling between oxygen uptake and ADP phosphorylation. Furthermore, inner mitochondrial membrane permeability, an important marker of the structural and functional integrity of this organelle, was evaluated by osmotic mitochondrial swelling.

The main purpose of this project was to evaluate the potential protective role of 660-nm laser light application to liver tissue by analyzing hepatic energy capacity based on previous studies from our research group concerning hepatic dysfunction, mitochondrial function and tissue levels of L-FABP. The liver consists of a complex organization of atoms that form molecules, structures, tissues and organs. Light has dual behaviors as a particle and a wave. The energy carried by light can be understood as an oscillation of electric and magnetic fields with no mass. These fields, when interacting with the charges of matter, cause matter to move by oscillation, especially electrons, which are lighter than atom nuclei, and create other electric and magnetic fields producing different effects, including electromagnetic waves 22. Some of these effects are more relevant to comprehension of the processes that occur when matter is illuminated, i.e., the energy transfer from light to molecules (absorption) and vice-versa (emission), and the changes in the course of light propagation (scattering). These three phenomena are very important to understanding the interactions between light and biological structures.

A fundamental concept in phototherapy is called biological optical window or therapeutic optical window, which represents the range of wavelengths that are most suitable for phototherapy applications when the abovementioned interactions are considered in biological tissues 23.

The results of the present study showed that prophylactic application of 660-nm laser light was able to minimize the harmful effects of ischemia and reperfusion, thus improving hepatic energy capacity, as observed by comparing the levels of oxygen consumption by hepatic mitochondria after the addition of ADP to the groups analyzed. Compared with the C group, a reduction in state 3 was observed in the PI group; however, no difference was found between the C and PIL groups, indicating that prophylactic laser application preserved mitochondrial energy functions.

This observation is supported by effects reported for other cells. Wong-Riley et al. 24 showed beneficial effects on neuron functionality after laser therapy at similar wavelengths. Eels et al. 25 observed similar effects for retinal injury, with preserved and stimulated mitochondrial function.

Changes in mitochondrial permeability include loss of inner mitochondrial membrane integrity due to gaps in nonspecific pores that release a flux of small molecules (<1500 Da) and protons. Consequently, significant mitochondrial swelling, loss of membrane potential, outer membrane rupture and apoptotic or necrotic death occur. Formation of these pores can occur in response to many stimuli, including Ca^+2^ overload and oxidative stress 15.

Studies of calcium- and phosphate-induced mitochondrial swelling have shown significant changes in internal mitochondrial membrane permeability in ischemic (PI) conditions, with no evidence of these changes in laser-treated ischemic groups (PLI). These results show that the IR process induced mitochondrial swelling with a change in membrane permeability, probably through interactions with ROS. However, this alteration was not sufficient to completely dissipate the electrochemical proton gradient since oxygen consumption velocity was maintained by the mitochondria in state 4.

Peng and Jou 26 showed how the interference of light in the blue-green range (488 nm) modifies ion balance, producing reactive oxygen species that open ion pores and changing the balance towards mitochondrial swelling. However, Shi et al. 27 showed that for IR wavelengths, laser irradiation can positively contributes to instead interfere with fragmented mitochondria recovery, including in liver cells, although laser-induced mitochondrial reactive oxygen species do not damage mitochondria through mitochondrial permeability transition pores. The transient opening of these pores may help release these species into the cytosol to protect the mitochondria against damage. This observation seems to corroborate the results obtained here.

The prophylactic use of laser light prevented mitochondrial swelling as evaluated using absorbance, reflecting an indirect measure of the degree of membrane damage, as shown in Figure 1, panel D. Studies have demonstrated that L-FABP is an effective marker of liver injury in various diseases, such as necrotizing enterocolitis, non-alcoholic fatty liver disease and liver tumors, and in liver transplantation 8,28,29. Due to its low molecular weight and high solubility, L-FABP can rapidly reach the bloodstream and be expressed in serum after the organ of origin has been damaged. L-FABP is found only in the cytoplasm of hepatocytes and, because these cells are in direct contact with the blood without an interstitial barrier, damage can cause its rapid release into the circulation 28. In the liver, inflammatory diseases that induce cytokine and bacterial factor release by Kupffer cells are known to decrease the expression of L-FABP in tissues. However, independent of these factors, simple rupture of the cellular barrier may be the cause of its rapid release 28. Akbal et al. 28 evaluated the serum levels of L-FABP in a clinical study of non-fatty alcoholic liver disease and found that it was an effective marker for early diagnosis of the disease. In an experimental study, Monbaliu et al. 7 demonstrated an increase in serum L-FABP in proportion to the time of exposure to hot ischemia in transplants performed in pigs. Other clinical studies have also demonstrated the efficacy of L-FABP as a biomarker of liver injury 30-32.

Our results were similar to those reported by Monbaliu et al. 7. We can hypothesize that L-FABP increased in serum as it decreased in tissues, although we did not measure L-FABP levels in plasma. A decrease in L-FABP was observed in hepatic tissues from the group submitted to PI, and a higher tissue level was observed in the group pre-conditioned with laser light (PLI); the results of the treated group and the control group were similar. The present results for tissue L-FABP expression are similar to those of Mitidiero et al. 8 who evaluated the tissue expression of L-FABP in necrotizing enterocolitis patients and found lower hepatic expression in this group. In the study, an inverse relationship was observed between intestinal fatty acid binding protein (I-FABP), a biomarker of intestinal tissue damage, and hepatic injury marked by L-FABP.

The effect of radiation in the far-red wavelengths of the spectrum on biological molecules has been reported in the literature. Cytochrome c oxidase (CcO) is reported to be a light absorber in mammalian cells, where it accelerates electron transfer reactions and increases ATP synthesis, ion antiporters and bomb activity. Light also dissociates nitric oxide (NO) bonds to CcO, increasing the respiratory rate and preventing NO-based cell death 32. Renno et al. 33 also showed that 660-nm light has regenerative effects, including stimulation of gene expression factors and ATP reactions. Although light can reportedly stimulate regeneration and the respiratory chain and prevent NO-based cell death, this stimulation has been reported to occur in tissues under regular conditions of temperature and blood flow. Ischemia and reperfusion are responsible for several sources of damage promotion in tissues, which are mediated by biochemical processes in which light can interfere. Therefore, these processes may interrupt the chain of biochemical responses to light stimulation. On the other hand, irradiation prior to ischemia may induce early activation of the biochemical chains that upregulate cell metabolism and activate regenerative processes in tissues. Thus, when damage actually occurs, these processes have already been initiated, which is probably the reason for the protective effect indicated by the increase in L-FABP expression.

Zhu et al. 34 showed that the same wavelength could be used to protect rat heart cells from damage similarly to liver cells, revealing that either pre- or post-ischemia irradiation can enhance tissue preservation. Later, Carnevalli et al. showed that IR laser therapy, in addition to establishing positive biomodulation, contributes to the reestablishment of cellular homeostasis in cells under nutritional stress and prevented apoptosis in animal ovarian (CHO K-1) cells 35. In the present study, we induced ischemia under normothermic conditions and found positive results for IR laser irradiation, demonstrating evidence of the favorable effect of light in the maintenance of tissues after damage-induced stress.

Therefore, the application of 660-nm laser light as a pre-conditioning treatment to the livers of rats submitted to partial ischemia was shown to be effective in protecting the liver, with L-FABP levels similar to those of controls.

Finally, redox-sensitive cellular targets have roles in cellular bioenergetics associated with changes of environmental stimuli, such as hormones, nutrients and oxygen tension, providing rapid and sensitive responses to changes in metabolism and fluxes in ROS. Conversely, ROS also have roles in cellular processes through redox-dependent signaling. In the present study, as expected, an increase in lipoperoxidation was observed with HIR (PI group), and the application of laser light was able to reverse or prevent ischemia-induced cellular changes, as shown by MDA levels, mitochondrial function parameters and L-FABP levels 36.

This project has limitations, including the lack of liver histology and immunohistochemistry analyses. Further studies are required to investigate L-FABP levels through these techniques and to study alternative pathways in liver injury to confirm the protective effects of laser light in hepatic ischemia.

## AUTHOR CONTRIBUTIONS

Vilalva KH performed the technical procedures, collected data and analyzed and interpreted the data in collaboration with Silveira M, Graf C and Mumic F. Figueira RL, Gonc�alves FL, Bagnato VS, D�Albuquerque LA, Sbragia L and Vollet-Filho JD were responsible for the intellectual and scientific content of the study. Gomes MC contributed to the bioenergetics evaluations. Castro e Silva O served as the mentor and tutor for the project and designed the protocol. Vilalva KH, Castro e Silva O, Figueira RL and Sbragia L were responsible for manuscript writing.

## Figures and Tables

**Table 1:** Malondialdehyde (MDA) and mitochondrial evaluation (State 3, State 4, RCR: Respiratory Control Ratio, Mitochondrial Swelling). The results are expressed as the mean ± standard deviation. C: Control; CL: Control + Laser; PI: Partial Ischemia; PIL: PI + Laser.

Group	MDA (µM/mg)	State 3 (nmol/ml)	State 4 (nmol/ml)	RCR (%)	Swelling (▵DO)
C	0.040 (±0.011)	83.35 (±15.12)	19.35 (±4.59)	4.30 (±0.22)	0.17 (±0.03)
CL	0.036 (±0.014)	97.13 (±12.74)	24.56 (±2.74)	3.88 (±0.22)	0.20 (±0.04)
PI	0.099 (±0.043)	66.27 (±17.45)	33.06 (±13.65)	2.56 (±1.49)	0.13 (±0.02)
PIL	0.037 (±0.029)	84.09 (±11.49)	29.59 (±13.42)	3.12 (±0.92)	0.14 (±0.02)
